# Host Factors Modulating Ochratoxin A Biosynthesis during Fruit Colonization by *Aspergillus carbonarius*

**DOI:** 10.3390/jof7010010

**Published:** 2020-12-28

**Authors:** Uriel Maor, Omer Barda, Sudharsan Sadhasivam, Yang Bi, Varda Zakin, Dov B. Prusky, Edward Sionov

**Affiliations:** 1 Institute of Postharvest and Food Sciences, The Volcani Center, Agricultural Research Organization, Rishon LeZion 7528809, Israel; uriel@volcani.agri.gov.il (U.M.); barda@volcani.agri.gov.il (O.B.); sudharsan@volcani.agri.gov.il (S.S.); veredz@volcani.agri.gov.il (V.Z.); dovprusk@volcani.agri.gov.il (D.B.P.); 2 Institute of Biochemistry, Food Science and Nutrition, The Robert H. Smith Faculty of Agriculture, Food and Environment, The Hebrew University of Jerusalem, Rehovot 7610001, Israel; 3 College of Food Science and Engineering, Gansu Agricultural University, Lanzhou 730070, China; beyang62@163.com

**Keywords:** OTA accumulation, sugar content, organic acids, pH modulation, fungal pathogenicity in fruits

## Abstract

*Aspergillus carbonarius* is a strong and consistent ochratoxin A (OTA) producer and considered to be the main source of this toxic metabolite in grapes and grape products such as wine, grape juice and dried vine fruit. OTA is produced under certain growth conditions and its accumulation is affected by several environmental factors, such as growth phase, substrate, temperature, water activity and pH. In this study, we examined the impact of fruit host factors on regulation and accumulation of OTA in colonized grape berries, and assessed in vitro the impact of those factors on the transcriptional levels of the key genes and global regulators contributing to fungal colonization and mycotoxin synthesis. We found that limited sugar content, low pH levels and high malic acid concentrations activated OTA biosynthesis by *A. carbonarius*, both in synthetic media and during fruit colonization, through modulation of global regulator of secondary metabolism, *laeA* and OTA gene cluster expression. These findings indicate that fruit host factors may have a significant impact on the capability of *A. carbonarius* to produce and accumulate OTA in grapes.

## 1. Introduction

Grapes are one of the largest produced fruit crops in the world. It is also one of the most abundant fruits: while almost 50% of grapes are used to make wine, one third is consumed as fresh fruit and the rest is dried, consumed as grape juice or stored in the form of grape musts [1]. Approximately 40% of table grapes are lost annually due to pathological and physiological deterioration [2], and in particular, because of the lack of efficient methods to prevent decay and senescence [3]. The black aspergilli (*Aspergillus* section *Nigri*) are frequently responsible for fungal decay of fresh fruit, and the consequent accumulation of mycotoxins. Among black aspergilli, *A. carbonarius* is a major producer of OTA in grapes, where it causes fungal decay and poses a significant threat to human health [4]. OTA contamination of grapes presents a serious health and economic problem, especially in Mediterranean countries [5,6]. OTA is a potent nephrotoxin that is responsible for several adverse effects on animals and humans; it has hepatotoxic, neurotoxic, carcinogenic, immunotoxic, genotoxic and teratogenic properties [7,8,9]. The International Agency for Research on Cancer (IARC) has classified OTA as a Group 2B possible human carcinogen, based on demonstrated carcinogenicity in animal studies [10,11]. Our recent study demonstrated that environmental pH plays an important role in *A. carbonarius* pathogenicity and OTA production [12]. Secretion of gluconic acid (GLA) by *A. carbonarius* caused direct fruit tissue acidification and induced accumulation of OTA in colonized grapes. Moreover, other environmental conditions, such as temperature, water activity and light, have been shown to contribute to OTA biosynthesis [13,14,15,16,17]. Composition of the growth medium has a strong effect on OTA production. Medina et al. [18] demonstrated that accumulation of the mycotoxin significantly altered under various carbon sources. The authors showed that the highest OTA levels produced by three *Aspergillus* isolates, *A. ochraceus*, *A. carbonarius* and *A. tubingensis*, were found in media supplemented with arabinose and glucose; fructose was the least favorable sugar for OTA production by the isolates. The same study, however, showed no marked difference in OTA production with regard to the nitrogen source. Compared to the extensive research on environmental factors that modulate mycotoxin formation, only a few studies were carried out to understand the effect of fruit host factors on mycotoxin biosynthesis. Fruit nutritional components that undergo changes during fruit maturation and ripening include sugars and organic acids, which may change fruit pH. This raises the question of whether such changes in the host can modulate fungal mycotoxin biosynthesis. Several studies on aflatoxin synthesis suggest that the high content of carbohydrates in figs, dates, citrus fruit and raisins probably enhances mycotoxin production by *Aspergillus flavus* and *Aspergillus parasiticus* [19,20]. It has been recently reported that apple host intrinsic factors, such as sucrose, organic acids and phenols, differentially modulate patulin accumulation during fruit colonization by *Penicillium expansum* [21]. In addition, several studies indicated that different apple and grape cultivars vary in their susceptibility to pathogen attack and mycotoxin accumulation in these fruits [22,23,24,25]. In the present work, we investigated the impact of grape intrinsic factors that change during fruit maturation on OTA accumulation in colonized fruits. Our results suggest that fruit host factors, including sugars, organic acids and pH, contribute to the complex activation of the global regulator of fungal secondary metabolism (LaeA) and OTA gene cluster, and may modulate the mycotoxin biosynthesis during fruit colonization by *A. carbonarius*.

## 2. Materials and Methods

### 2.1. Fungal Strain and Culture Conditions

The *A. carbonarius* NRRL 368 strain used throughout this study was obtained from USDA Agricultural Research Service Culture Collection (Northern Regional Research Laboratory, Peoria, IL, USA). The isolate was stored in 25% glycerol at −80 °C until use and maintained on potato dextrose agar (PDA) plates (BD, Franklin Lakes, NJ, USA). Conidia were harvested with 10 mL of sterile saline and adjusted using an hemocytometer to the indicated concentrations. A 10^6^ fungal spores/mL solution (100 µL) was inoculated onto 55 mm petri dishes with 10 mL of solid synthetic grape juice media (SGM; [26]) containing (per liter): 0.67 g (NH_4_)_2_HPO_4_, 0.67 g KH_2_PO_4_, 1.5 g MgSO_4_·7H_2_O, 0.15 g NaCl, 0.15 g CaCl_2_, 0.0015 g CuCl_2_, 0.021 g FeSO_4_·7H_2_O, 0.0075 g ZnSO_4_·7H_2_O, different sugar concentrations (10, 50, 100 and 150 g/L, as indicated in each experiment)—glucose-fructose at a ratio of 2.33 (70% D(+)glucose and 30% D(−)fructose), different concentrations of malic acid (10, 20, 30 and 40 g/L), different concentrations of tartaric acid (0, 3.5, 7 and 14 g/L), various concentrations of hydrated catechin (0, 0.025, 0.05 and 0.1 g/L) and 2% agar adjusted to different pH levels (3, 4 and 5) with concentrated KOH. The plates were incubated at 28 °C in the dark for 2–8 days as needed for sample collection.

### 2.2. Fruit Colonization and Pathogenicity Experiments

White “Superior” grapes were freshly harvested from two trees in a single vineyard in central Israel (Moshav Pedaya) during two consecutive years, approximately 30 days after fruit set (early harvest). Four additional harvests were made within 10 days intervals. For each harvest, five bunches containing 20–30 berries in each bunch were collected. The grape berries were homogeneous in size and color, and had no visible damage or symptoms of fungal attack on the skin. Harvested fruits were analyzed for acidity, pH and TSS as described below. On the day of harvest, berries in the same bunch were submerged in 1% sodium hypochlorite solution for 1 min and immediately rinsed twice in sterile distilled water for surface sterilization. The sterilized grapes were aseptically air-dried and inoculated by injection with 10 μL of *A. carbonarius* conidial suspension (10^6^ spores/mL) onto each grape berry. Following inoculation, the fruits were incubated under high humidity at 28 °C for 7 days as needed for symptom monitoring and sample collection, and the diameters of the rotten spots were recorded daily.

### 2.3. pH Measurements, Organic Acids and OTA Analysis

pH was measured directly in the agar cultures with a double pore slim electrode connected to a Sartorius PB-11 Basic Meter (Sartorius, Goettingen, Germany). For assessment of GLA production, five 1-cm diameter discs of agar were placed in 5 mL of sterilized water and crushed to homogeneity. A 1 mL aliquot of the solution was sampled in a 1.5 mL microcentrifuge tube and centrifuged for 10 min at 20,800× *g*. The supernatant was taken for GLA analysis using a test kit applying an enzymatic method for the specific measurement of total D-Gluconic acid content (Megazyme, Wicklow, Ireland) according to the manufacturer’s instructions. To evaluate OTA levels, five 1-cm diameter discs of agar were added to 1.7 mL of HPLC grade methanol (Bio-Lab, Jerusalem, Israel) and crushed to homogeneity. OTA was extracted by shaking for 30 min at 150 RPM on an orbital shaking platform and centrifuged for 10 min at 20,800× *g*. The supernatant was filtered through a 0.22 µm PTFE syringe filter (Agela Technologies, Tianjin, China) and kept at −20 °C prior to HPLC analysis. OTA was quantitatively analyzed by injection of 20 µL into a reverse phase UHPLC system (Waters ACQUITY Arc, FTN-R, Milford, MA, USA). The mobile phase consisted of acetonitrile:water:acetic acid (99:99:2, *v*/*v*/*v*) at 0.5 mL/min through a Kinetex 2.6 µm XB-C18 (100 mm × 2.1 mm) with a security guard column C18 (4 mm × 2 mm) (Phenomenex, Torrance, CA, USA). The column temperature was maintained at 30 °C. The OTA peak was detected with a fluorescence detector (excitation at 330 nm and emission at 450 nm) and quantified by comparing with a calibration curve of the standard mycotoxin (Fermentek, Jerusalem, Israel).

The pH of grape tissues was measured by inserting a double pore slim electrode directly into the tested area. For assessment of malic acid content in freshly harvested grapes and GLA production in colonized grape tissue, the berries were crushed manually in 50 mL tubes; the juice was recovered after centrifugation and was taken for analysis of these organic acids. GLA and malic acid concentrations in grapes were determined by using enzymatic methods for measurement of total GLA/malic acid content (Megazyme, Wicklow, Ireland) according to the manufacturer’s instructions. The TSS content in grape juice was measured by a digital refractometer (Atago, Tokyo, Japan). For OTA analysis in colonized grapes, 1.7 g of the macerated necrotic area was taken, 1.7 mL of HPLC grade methanol (Bio-Lab, Jerusalem, Israel) was added and the tissues were homogenized. Then, OTA was quantitatively analyzed as described above.

### 2.4. RNA Extraction and Gene Expression Analysis by qRT-PCR

Mycelia from the in vitro experiments were harvested at the appropriate time, weighed, frozen in liquid nitrogen, lyophilized for 24 h and kept at −80 °C until used. Total RNA was extracted from 100 mg of ground mycelium of the selected samples using the Hybrid-R RNA isolation kit (GeneAll, Seoul, Korea) according to the manufacturer’s protocol. The DNase and reverse-transcription reactions were performed on 1 µg of total RNA with the Maxima First-Strand cDNA Synthesis Kit (Thermo Scientific, Waltham, MA, USA) according to the manufacturer’s instructions. The cDNA samples were diluted 1:10 (*v*/*v*) with ultrapure water. The quantitative real-time PCR was performed using Fast SYBR green Master Mix (Applied Biosystems, Waltham, MA, USA) in a StepOnePlus Real-Time PCR System (Applied Biosystems, Waltham, MA, USA). The PCR conditions were as follows: 95 °C for 20 s, followed by 40 cycles of 95 °C for 3 s and 60 °C for 20 s. The samples were normalized using *β-tubulin* as endogenous control and the relative expression levels were measured using the 2^(−ΔΔCt)^ analysis method. Results were analyzed with StepOne software v2.3. Primer sequences used for qRT-PCR analysis are listed in Table S1.

### 2.5. Statistical Analysis

Student’s *t*-test was performed when data was normally distributed and the sample variances were equal. For multiple comparisons, one-way ANOVA was performed when the equal variance test was passed. Significance was accepted at *p* < 0.05. All experiments described here are representative of at least three independent experiments with the same pattern of results.

## 3. Results and Discussion

### 3.1. Fruit Maturation Affects OTA Production by A. carbonarius

Several physiological parameters of freshly harvested white “Superior” grapes were measured at different periods during fruit maturation (Table 1). A gradual increase in the total soluble solids (TSS) of grape berries was observed during fruit maturation. TSS of the fruits from the 5th latest harvest time point increased by 11.01% to 14.98%, compared to 3.97% of that from the 1st “early” grape harvest time point. Furthermore, comparison of “early” and “late” maturity harvested fruits showed a decrease by 2.08% in malic acid (from 3.16% to 1.08%), which is the main organic acid found in grapes, and an increase in pH values from 2.96 to 4.61 in more mature fruits (Table 1).

**Figure 1:**
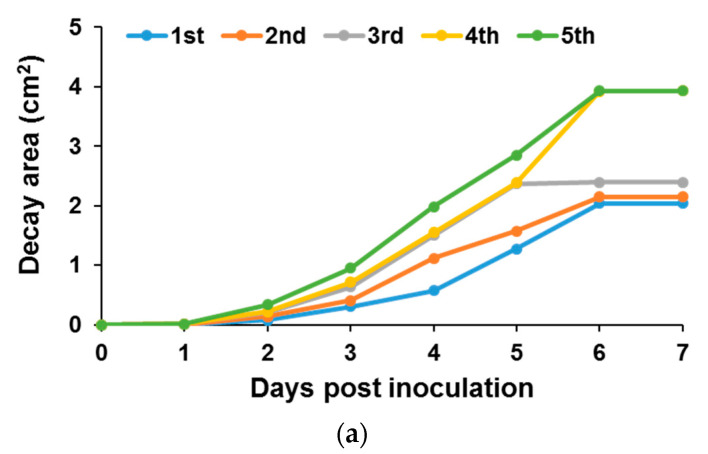

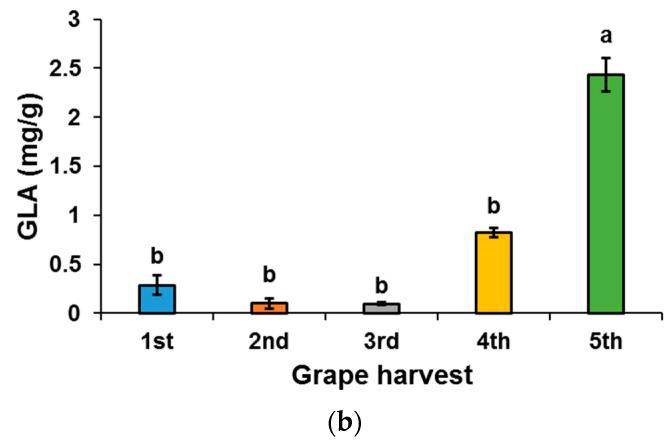

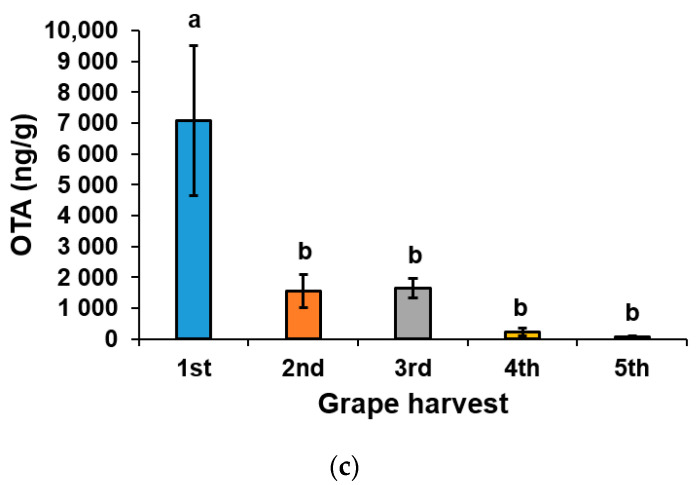
Disease development in freshly harvested fruits at different time points following the inoculation of “Superior” grape berries by *A. carbonarius*. The grape berries were inoculated immediately after harvest. (**a**) Histogram showing the decay area of the rotten tissue on infected grape berries. (**b**) GLA and (**c**) OTA accumulations were measured in infected fruits at day 7 post-inoculation. Error bars represent standard error of three independent biological replicates. Different letters above the columns indicate statistically significant differences at *p* < 0.05, as determined using the Tukey’s honest significant difference test.

To determine the effect of these dynamic changes on OTA accumulation by *A. carbonarius*, grape berries were inoculated at different harvest periods during fruit maturation (Figure 1).

Fungal infection caused a significant increase in the rotten colonized area of the late harvested fruits (4th and 5th harvests) relative to that of the early harvested grape berries (Figure 1a). Disease development was accompanied by accumulation of relatively high amounts of GLA in more mature grape berries (Figure 1b). One of the factors that contribute to the pathogenicity of *A. carbonarius* is its ability to produce GLA [12,27]. GLA accumulation by *A. carbonarius* is carried out by glucose oxidase that catalyzes the oxidation of glucose to GLA. Recently we have provided evidence that deletion of the glucose oxidase-encoding gene, *gox*, in *A. carbonarius* led to a reduction in virulence toward nectarine and grape fruits, further indicating that GOX is a virulence factor of *A. carbonarius* [28]. Interestingly, an opposite trend was observed regarding the accumulation of OTA in grapes harvested at different time points. A high amount of OTA was found to be produced by *A. carbonarius* in early harvested low sugar grapes (1st harvest, 7084 ng OTA per gram of grape berries), compared to significantly lower concentrations of the mycotoxin accumulated in grape berries with increased maturity at 4th and 5th harvests (241.1 ng/g and 71.3 ng/g, respectively) (Figure 1c). Several host factors may influence the mycotoxin biosynthesis by the fungus during the early period of grape berry growth. A relatively high concentration of malic acid may trigger excessive OTA production by the fungus in early harvested grape berries. Malic acid levels decrease during grape ripening. A reduction in malic acid content was also observed in late maturity harvested apples compared to early harvested fruits [21]. In this study, however, a relatively high amount of patulin was observed in more mature apples colonized by *P. expansum*, indicating complex host–pathogen interactions in different fruits.

### 3.2. Association of Sugar Content and pH with OTA Biosynthesis

**Figure 2:**
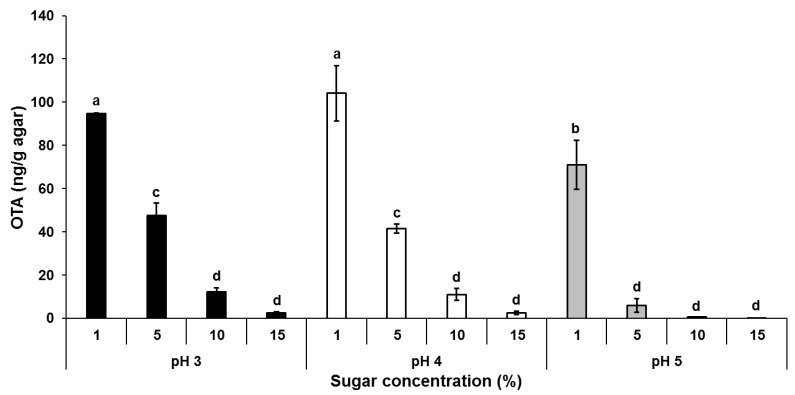
OTA biosynthesis is modulated by sugar and pH levels. Solid synthetic grape juice media (SGM) was supplemented with different sugar concentrations (glucose/fructose at a ratio of 2.33), and inoculated with *A. carbonarius* (10^6^ spores/mL). OTA accumulation was evaluated at day 6 post-inoculation. Error bars represent standard error of three independent biological replicates. Different letters above the columns indicate statistically significant differences at *p* < 0.05, as determined using the Tukey’s honest significant difference test.

Considering the high concentration of OTA produced in early harvested grapes and a reduced amount of the mycotoxin in more mature fruits with increasing TSS and pH values (Table 1, Figure 1), we sought to determine the relationship between sugar concentration, pH and OTA production. *A. carbonarius* was grown on solid artificial SGM medium amended with increasing sugar concentrations (from 1 to 15%) of glucose–fructose combination, under different pH levels (3, 4 and 5). At day 6 post-inoculation, OTA levels were maximal at 1% sugar concentration under initial pH 4.0, reaching 104 ng/g agar. This amount decreased with increasing sugar concentration, reaching a minimum of 2.6 ng/g in the presence of 15% sugar (Figure 2). These results point out to the same trend of OTA accumulation observed in vivo during grape berries maturation and ripening.

**Figure 3:**
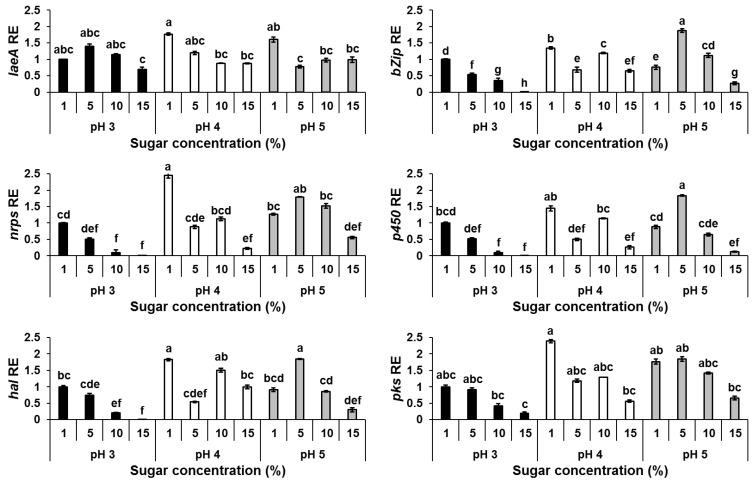
*laeA* and OTA gene cluster relative expression as affected by sugar concentrations and pH levels. Solid SGM media was amended with different sugar concentrations. The media was inoculated with 100 µL of a 10^6^ spore/mL suspension and the relative expression of *laeA*, *bZIP*, *nrps*, *p450*, *hal* and *pks* genes were compared at different conditions. RNA was extracted from mycelia at day 6 post-inoculation. Relative expression was normalized using β-tubulin as an internal control. The expression of each gene under sugar concentration of 1% at pH 3 was normalized as 1.0. Error bars represent standard error of three independent biological replicates. Different letters above the columns indicate statistically significant differences at *p* < 0.05, as determined using the Tukey’s honest significant difference test.

To investigate the influence of sugar concentration and ambient pH upon OTA production at the molecular level, we analyzed the differential expression of the *laeA* gene, encoding a methyltransferase involved in global regulation of fungal secondary metabolism, and OTA biosynthetic cluster genes in *A. carbonarius* under different physiological conditions. As shown in Figure 3, the expression levels of the global regulator *laeA* and all five genes involved in OTA biosynthesis correlated with mycotoxin production under different sugar concentrations. The increased OTA accumulation that occurred under limited sugar concentrations of 1% and 5% was accompanied with *laeA* and almost all OTA biosynthesis cluster genes being upregulated, while a reduction of the mycotoxin production accompanied with decreased relative expression of the genes was observed in the presence of higher sugar concentrations (Figure 2 and Figure 3).

Our findings are consistent with a previous study of Kumar et al. [21], which reported repression in relative expression of *laeA*, patulin biosynthesis cluster genes and patulin production by *P. expansum* in the presence of increased sucrose concentrations in synthetic growth medium, whereas maximal gene expression and patulin accumulation were obtained when the fungus was grown at a limited sucrose level.

Another factor that might affect OTA accumulation during fruit maturation is pH change as it was found that pH levels in the fruit increased with grape berries maturation from 2.96 to 4.61 (Table 1). A number of studies have reported that OTA biosynthesis increases in *Aspergillus ochraceus* when the pH drops below 6.0 [29,30]. Recently, we found that OTA production by *A. carbonarius* is also strongly influenced by ambient pH with higher amounts of the mycotoxin being produced under acidic pH (3.0–4.0) than at neutral or alkaline conditions [12,27]. Our in vitro experiments confirmed that an acidic environment is favorable for OTA production, with increased mycotoxin synthesis observed at pH 3 and 4, but only under a limited sugar content (Figure 2). pH changes in the presence of increased sugar concentrations from 1 to 15% also affected *laeA* and OTA biosynthesis gene expression (Figure 3).

The growth substrate is one of the most important factors influencing mycotoxin biosynthesis. High sucrose content as a carbon source in defined media (such as YES—yeast extract sucrose) strongly influences OTA production in *A. ochraceus*, *Aspergillus sclerotiorum*, *Aspergillus sulphurus*, *Penicillium verrucosum* and *Penicillium nordicum* [31,32]. YES medium containing 15% sucrose was shown to be highly supportive for OTA biosynthesis by *A. carbobarius* under acidic pH [12]. This increase in OTA was accompanied by induction of the transcript levels of *laeA* and OTA biosynthesis genes under high sucrose concentration compared with that in the sucrose-limited medium. Interestingly, OTA biosynthesis is strongly inhibited in media containing glucose as sole carbon source [33]. *P. expansum* produced less patulin when grown in the presence of glucose and fructose compared to sucrose-amended medium [34]. According to the results obtained in the current study, this seems to be the case where high concentrations of glucose and fructose, which are the main sugars in grape berries, inhibit OTA production. Secondary metabolite biosynthesis in many filamentous fungi, including *A. carbonarius*, is tightly regulated by both the pathway-specific transcription factor embedded in mycotoxin biosynthesis gene cluster (e.g., bZIP transcription factor in the OTA cluster) and by global regulatory factors, including the global regulator of secondary metabolites (LaeA), the pH regulatory factor PacC and the carbon catabolite repressor (CreA) [27,35,36,37]. Previous studies have reported that involvement of global carbon catabolite regulator CreA in secondary metabolism may reflect the differences seen in metabolite production when fungi are grown in different carbon sources [38,39]. In the present study, a significant upregulation of *creA* gene was found under high sugar content (Figure S1), suggesting that CreA is involved in OTA synthesis, possibly through the negative regulation of LaeA and the OTA gene cluster. These results are supported by some previous studies that found negative correlation between transcript level of secondary metabolites biosynthesis genes and *creA* expression in *Penicillium chrysogenum*, *Penicillium oxalicum* and *P. expansum* [34,39,40].

### 3.3. Malic Acid Modulates laeA Expression and OTA Biosynthesis

**Figure 4:**
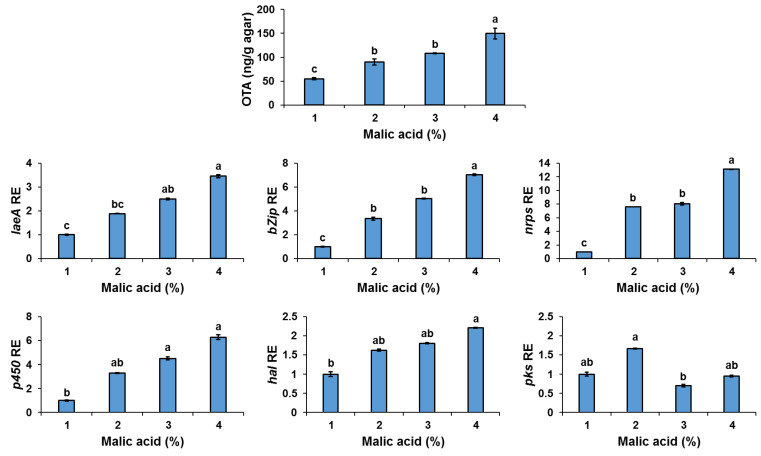
Effect of malic acid on OTA synthesis by *A. carbonarius* in SGM media. Solid SGM-media was supplemented with 1–4% concentrations of malic acid and inoculated with 100 µL of a 10^6^ spore/mL. OTA accumulation and relative expression of *laeA* and OTA gene cluster were evaluated 6 days post-inoculation. Relative expression was normalized using β-tubulin as an internal control. Error bars represent a standard error of three independent biological replicates. Different letters above the columns indicate statistically significant differences at *p* < 0.05, as determined using the Tukey’s honest significant difference test.

Given that the presence of high concentration of malic acid in early harvested grape berries is correlated with increased OTA production during colonization by *A. carbonarius*, we examined the influence of this organic acid on OTA biosynthesis at the molecular level. Figure 4 shows that increasing the concentrations of malic acid from 1 to 4% in the media, led to a 3.5-fold upregulation of *laeA* and up to a 13-fold increase in OTA cluster gene expression, accompanied by an elevation in OTA biosynthesis from 54 to 150 ng/g agar.

**Figure 5:**
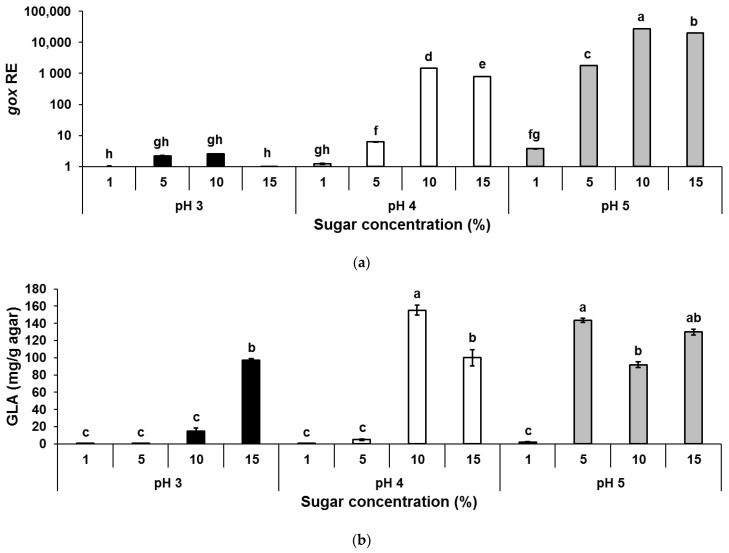
Effect of different sugar concentrations and pH changes on GLA accumulation and relative expression of *Acgox* gene in vitro. Solid SGM media was supplemented with different sugar concentrations (glucose/fructose at a ratio of 2.33), and inoculated with 100 µL of a 10^6^ spores/mL suspension. (**a**) *Acgox* relative expression, and (**b**) GLA accumulation were analyzed 6 days post-inoculation. Relative expression was normalized using β-tubulin as an internal control. The expression of *gox* gene under a sugar concentration of 1% at pH 3 was normalized as 1.0. Error bars represent a standard error of three independent biological replicates. Different letters above the columns indicate statistically significant differences at *p* < 0.05, as determined using the Tukey’s honest significant difference test.

These findings align well with a previous study, where increasing concentrations of malic acid enhanced activation of *laeA* and patulin synthesis by *P. expansum* in vitro [21]. The activation of the global regulator LaeA and OTA gene cluster, and consequently increased OTA accumulation, was probably caused due to the acidification process induced by malic acid. Previous findings indicate that acidification of the host environment through accumulation of organic acids enhanced fruit maceration and colonization by fungal pathogens [12,27,28,41,42,43]. These studies point out the importance of the acidification process driven by the accumulation of another organic acid produced by *P. expansum* and *A. carbonarius*, GLA, which contributes to enhanced fungal pathogenicity in vivo and mycotoxin accumulation. Indeed, accumulation of high amounts of GLA was found in the colonized grape berries at a late maturity stage (Figure 1b) with high TSS content and relatively higher pH levels compared to those parameters of the early harvested grapes (Table 1). These results correlate with our in vitro experiments, where a significant upregulation of the *gox* gene expression accompanied by increased production of GLA, was observed under high sugar contents at rising pH levels (Figure 5a,b). However, mycotoxin production was minimal under these conditions both in vitro and during colonization of late harvested grapes that contain lower amounts of malic acid. There is no clear explanation on the importance of malic acid on the regulation of OTA. Malic acid belongs to a group of C_4_ dicarboxylic acids that are produced by many microorganisms as key intermediates of the tricarboxylic acid (TCA) cycle in primary metabolism. While degradation of malic acid during grape ripening is well known, it is still not clear how this process modulates any biochemical pathways that may affect OTA synthesis.

### 3.4. The Role of Other Grape Nutrition Factors in OTA Biosynthesis

The influence of another two key nutrient factors in grapes, tartaric acid and catechin, on OTA synthesis was examined in the current study. Grapes are one of the few fruits to contain tartaric acid. Tartaric acid accumulates in grape berries along with malic acid during the first period of the grapes’ growth; these acids are responsible for the acidity of the future wine. During the ripening stage, malic acid is metabolized and used as an energy source; its proportion decreases toward the tartaric acid production, which remains almost unchanged [44]. The concentration range of tartaric acid found in grapes is 1.28–7.45 g/L [45]. In our in vitro experiments, no significant change in OTA accumulation was observed in the presence of increasing tartaric acid concentrations in solid media from 0 to 14 g/L (Figure S2a). These results suggest that, unlike malic acid, tartaric acid is not associated with OTA biosynthesis.

Catechin is one of the main phenolic compounds in grape seeds and skins, with amounts ranging between 74 and 205 mg/100 g of dry matter [46]. This compound is proven to be a potent antioxidant and to possess important biological, pharmacological and medicinal properties [47]. Similarly to the experiment with tartaric acid, supplementation of different concentrations of catechin to the SGM media did not affect mycotoxin production by *A. carbonarius* (Figure S2b), suggesting that there is no evidence for the involvement of this phenolic compound in the modulation of OTA synthesis. These results are in contrast with those reported previously by Kumar et al. [21], where phenolic compounds like chlorogenic acid and epicatechin, were shown to differentially modulate *laeA* expression and patulin synthesis in *P. expansum*. Moreover, other studies demonstrated antifungal and antimycotoxigenic activities of phenolic compounds in several agricultural commodities [48,49]. We assume that, in general, one would expect large differences in the response of different fungal pathogens to phenolic compounds, mostly determined by the fungal genus and/or specific host.

## 4. Conclusions

This study was important in improving our basic understanding of the mechanisms by which a number of the grape key host factors may affect mycotoxin accumulation during fruit colonization. Our findings strongly suggest that a series of intrinsic critical host factors that change during fruit ripening, including sugars, organic acids and pH, have a significant impact on *A. carbonarius* capability to produce OTA in grapes. A strong in vivo–in vitro correlation was observed throughout the study, where limited sugar and low pH levels but high malic acid concentrations activated OTA biosynthesis by the fungus both in synthetic media and during fruit colonization through modulation of *laeA* and OTA gene cluster expression. Regulation of secondary metabolism by fruit host factors could serve as a model system for understanding the pH-regulating processes in fungal pathogens. In addition, modulation of fruit sugar content and/or fruit tissue pH during disease development might enable designing control measures that will focus on mitigating the consequences of the pathogen invasion.

## Figures and Tables

**Table 1:** Physiological parameters of harvested fruits.

Grape Harvest	TSS (%)	Fruit pH	Malic Acid (%)
1st	3.97 ± 0.15 (e)	2.96 ± 0.15 (e)	3.16 ± 0.06 (a)
2nd	5.60 ± 1.29 (d)	3.16 ± 0.14 (d)	3.22 ± 0.18 (a)
3rd	7.40 ± 1.26 (c)	3.46 ± 0.21 (c)	2.71 ± 0.22 (b)
4th	13.44 ± 1.25 (b)	4.08 ± 0.19 (b)	1.36 ± 0.07 (c)
5th	14.98 ± 0.78 (a)	4.61 ± 0.25 (a)	1.08 ± 0.05 (c)

Total soluble solids (TSS), pH values of harvested grapes and concentration of malic acid in grapes at different time points. The harvests were made within a 10 days interval. For each harvest, five bunches containing 20–30 berries in each bunch were collected. Different letters in parentheses indicate statistically significant differences at *p* < 0.05, as determined using the Tukey’s honest significant difference test.

## Supplementary Materials

The following are available online at https://www.mdpi.com/2309-608X/7/1/10/s1, Table S1: Primer sequences used for qRT-PCR analysis; Figure S1: Effect of sugar concentrations and pH changes on the relative expression *creA* gene; Figure S2: Effect of tartaric acid and catechin on OTA accumulation in vitro.
