# Supplementary material for: Host Factors Modulating Ochratoxin A Biosynthesis during Fruit Colonization by Aspergillus carbonarius
Source: J Fungi (Basel). 2020 Dec 28;7(1):10. doi: 10.3390/jof7010010 (PMC7823970; doi:10.3390/jof7010010)
Supplement: Supplementary file 1 [file jof-07-00010-s001.pdf]

Supplementary material

Table S1. Primer sequences used for qRT-PCR analysis

#	Primer name	Sequence (5'-3')	Description
1	Btub_carF	GGACGAGATGGAGTTCACTGA	*β-tubulin* control gene
2	Btub_carR	CCTCTTGCTCAAGGACCTCCT	
3	AcLaeA LI	CACCTATACAACCTCCGAACCAC	Velvet complex *laeA* gene
4	AcLaeA LJ	GGTTCGGCCAACCGACGACGCTG	
5	AcGox f2	TCGAACACTCTGGCATTGGA	Glucose oxidase (*gox*) gene
6	AcGox r2	GTGGTTTGGTCCTGCAGGTT	
7	PKS_carF	GGGATCGTACGATCTGGTGAT	OTA cluster polyketide synthase
8	PKS_carR	GGGAACACATGAGGTCAGGCT	
9	NRPS_carF	CGGTAGAAAGACTGCAGTCCAT	OTA cluster non-ribosomal peptide synthetase
10	NRPS_carR	CGTCGGAATCCATTGCGCTGA	
11	bZip_carF	CTCGACGGTTCGAGCCTTCT	OTA cluster bZip transcription factor
12	bZip_carR	GCATTCGCTCTAGCTGCTCGA	
13	P450_carF	CCATCGTCTCCAGAGAATCAGT	OTA cluster cytochrome P450 monooxygenase
14	P450_carR2	GGTCTCGTCGTGATGAATCAAG	
15	HAL_carF	GCCAGTAGAGGGACAGCCAT	OTA cluster halogenase
16	HAL_carR	GCTGGAGGTGGTGGTTGAGA	
17	AcCreA f1	CCCTCAGGTGCCGAAAGTC	Carbon catabolite repressor *creA* gene
18	AcCreA r1	CGAAGTCACCGAGGAAAACC	

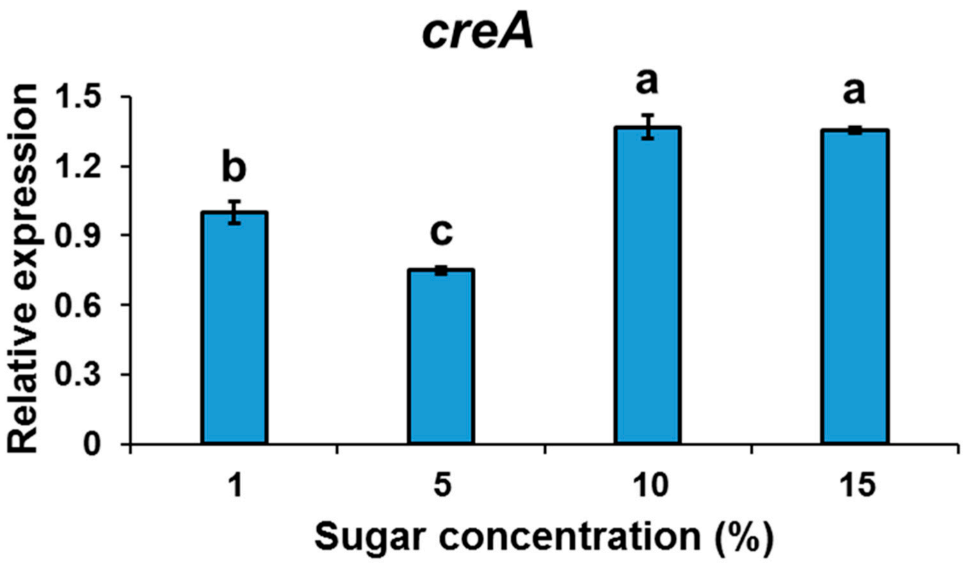

Figure S1. Effect of sugar concentrations and pH changes on the relative expression *creA* gene. Solid SGM media was supplemented with different sugar concentrations (glucose/fructose at a ratio of 2.33). The media was inoculated with 100 µl of a 10^6 spores/ml *A. carbonarius* suspension and the relative expression of *creA* was compared at different conditions. RNA was extracted from mycelia at day 6 post-inoculation. Relative expression was normalized using *β-tubulin* as an internal control. The gene expression under sugar concentration of 1% was normalized as 1.0. Error bars represent standard error of three independent biological replicates. Different letters above the columns indicate statistically significant differences at $p<0.05$, as determined using the Tukey's honest significant difference test.

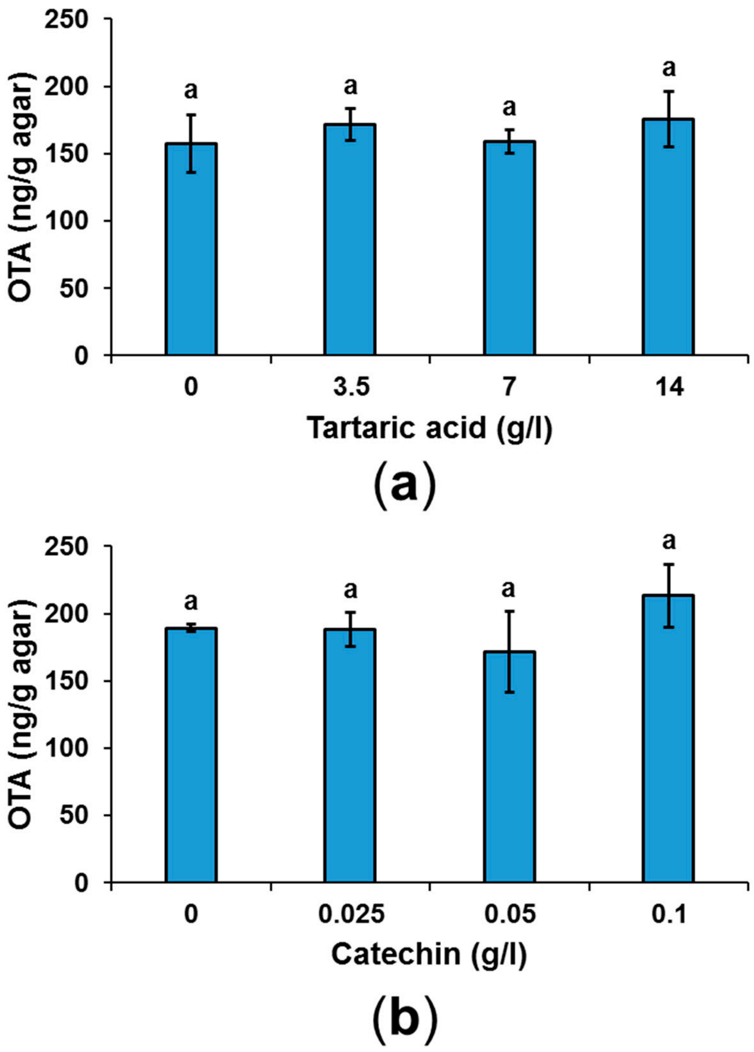

Figure S2. Effect of tartaric acid and catechin on OTA accumulation *in vitro*. Solid SGM media was amended with various concentrations of tartaric acid and catechin. Then, the media was inoculated with 100 µl of a 10^6 spore/ml suspension of *A. carbonarius*, and OTA accumulation was evaluated at day 6 post-inoculation under different concentrations of tartaric acid (**a**) and catechin (**b**). Error bars represent standard error of three independent biological replicates. The letters above the columns indicate no statistical difference, as determined using the Tukey's honest significant difference test.
